# Microstructure Evolution and Plastic Deformation Mechanism of Cold Rolling Deformation of Micro/Nano Pure Electrolytic Nickel

**DOI:** 10.3390/ma19020235

**Published:** 2026-01-07

**Authors:** Han Zhang, Jisen Qiao, Hao Yang, Yangtao Xu, Tiandong Xia

**Affiliations:** 1 School of Materials Science and Engineering, Lanzhou University of Technology, Lanzhou 730050, China; 18793138809@163.com (H.Z.); hal.yang@vatgroup.com (H.Y.); lanzhouxuyt@163.com (Y.X.); 2 State Key Laboratory of Advanced Processing and Recycling of Non-Ferrous Metal, Lanzhou University of Technology, Lanzhou 730050, China

**Keywords:** electrolytic nickel, cold rolling, plastic deformation mechanism, detwinning, annealing twins

## Abstract

This paper investigates the cold rolling (CR) deformation behavior of electrolytic nickel at room temperature. While the microstructural evolution across deformation levels ranging from 5% to 98% is systematically characterized. The deposited electrolytic nickel exhibits numerous growth twins of various lengths and thicknesses, accounting for over 70% of the microstructure. The average grain size is 0.56 μm, and the grain size distribution is relatively broad. The plastic deformation of electrolytic nickel in the early stage is governed by the interaction between high-density dislocations and abundant twins. The primary mechanism accommodating deformation is detwinning. At 70% deformation, under high strain, complete detwinning occurs. When the CR reaches 90%, the average short-axis grain size is refined to 113 nm, indicating the deformation-induced refinement limit of electrolytic nickel. The microstructure at this stage exhibits a typical lamellar morphology. At 98% deformation, the average microhardness peaks at 240.3 HV, representing a cumulative increase of 46.88%. Dynamic recovery and recrystallization are observed at both 70% and 98% deformation levels, accompanied by the formation of Σ3 {120} type incoherent twins within recrystallized grains. Under large strain, the dominant cold plastic deformation mechanism transitions to a synergistic effect of dislocation slip and stratification.

## 1. Introduction

With the rise of new energy vehicles, high-purity nickel [1] has found widespread applications in industries such as sputtering targets [2,3], electrical equipment, and power batteries due to its excellent electrical conductivity, outstanding corrosion resistance [4], and superior strength and plasticity [5,6,7]. Currently, the industrial production of nickel processing materials involves remelting electrolytic nickel into ingots, followed by multiple hot and cold processing steps. This method is complex, energy-intensive, and highly polluting [8,9]. New impurities are introduced during the casting process [10,11], which negatively impacts the purity of the metal [12,13]. To address these challenges, short-process rolling technology [14] has been proposed to directly roll electrolytic nickel [15,16] and produce related processing materials. This approach preserves the high purity of the metal, reduces internal resistance, and shortens both the production cycle and costs.

Dislocation slip, twinning, and shear are three primary mechanisms of plastic deformation in metallic materials [17,18,19]. In recent decades, extensive research has been conducted on the preparation, microstructure, mechanical properties, and deformation mechanisms of electrodeposited metallic materials [20,21,22,23,24,25,26,27,28]. Pure nickel, a face-centered cubic metal with medium to high dislocation energy, typically undergoes plastic deformation primarily via dislocation slip [20]. However, several fundamental issues remain unresolved, such as grain boundary structure, defect movement, and deformation and fracture mechanisms. Moreover, most studies have focused on laboratory-deposited pure nickel thin sheets or films [21,22], which exhibit limited ductility and deformation capacity. The strain observed in experiments is restricted, and the experimental observations are confined to a small region.

Electrolytic nickel, with its high density and purity, has a unique microstructure compared to traditional coarse-grained materials [23,24,25]. For this complex and structurally distinctive electrolytic nickel material, it is essential to understand the evolution of its microstructure and mechanical behavior during plastic deformation. Therefore, this study focuses on electrolytic nickel, which is directly rolled at room temperature to systematically investigate the effects of deformation amount and initial structure on the microstructural evolution during the cold rolling (CR) deformation process. The deformation mechanism during cold plastic deformation is also examined. By studying the crystal structure transformation of electrolytic nickel during cold plastic deformation, this research provides a necessary theoretical basis for the direct rolling of electrolytic metal materials. This work is of great significance for optimizing short-process rolling technology and enhancing the overall performance of nickel strip products.

## 2. Materials and Methods

The experimental material used in this study was industrial electrolytic nickel (Jinchuan Group Co., LTD, Jinchang, China). Electrolytic nickel is produced by melting and casting nickel sulfide to form soluble anode plates of specified specifications. These plates are then electrolytically refined in a sulfate solution, with the nickel being deposited on high-purity nickel starting sheets on both sides, ultimately yielding electrolytic nickel products of various specifications [26]. The thickness of the deposition varies with the deposition time, reaching up to 20 mm at its thickest. The main chemical composition of the electrolytic nickel is presented in Table 1 [27].

**Figure 1:**
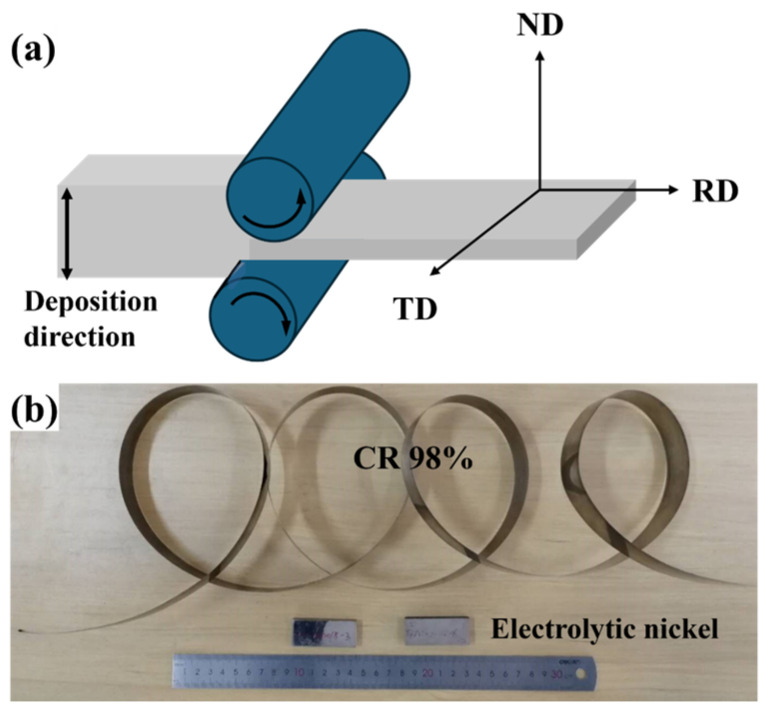
Shows the schematic diagram of cold rolling and the physical sample: (**a**) Illustration of the cold rolling process; (**b**) Electrolytic nickel rolling samples.

The electrolytic nickel was processed into 50 mm × 20 mm × 9 mm samples using wire cutting (DK7725E, AVIC Changfeng Numerical Control Technology Co., LTD., Suzhou, China). After surface grinding, the samples were placed in a Φ350 × 400 two-high rolling mill (Wuxi Guancheng Metal Technology Co., LTD., Wuxi, China) for multiple CR passes. The rolling process and the samples are shown in Figure 1. The normal direction (ND) of the rolling surface is aligned with the direction of the electrolytic nickel deposition. The deformed samples are labeled by their degree of rolling deformation, which includes: CR 5%, CR 10%, CR 30%, CR 70%, CR 90%, and CR 98%. The rolling speed was set between 0.1 to 0.2 m/s, and no intermediate annealing treatment was applied during the rolling process.

The hardness of the samples was measured using the Wilson 1102D37 (Buehler Ltd., Lake Bluff, IL, USA) fully automatic microhardness tester (American Standard). Wire cutting was employed to extract samples from the cross-section of the nickel plate, ensuring that the upper and lower surfaces were parallel. The test load was set at 0.2 kgf, with a loading time of 12 s.

After the samples were sanded with sandpaper and electrolytically polished, the microstructure of the rolling direction (RD) surface was examined using the Oxford-SYMMETRY S3 electron back-scattered diffraction (EBSD) system (Oxford Instruments, Abingdon, Oxfordshire, UK). The EBSD data collection parameters, including the area and step size, were set based on the microstructural state of the sample under different conditions. Electrolytic polishing was performed using a 5% perchloric acid + 95% alcohol solution at room temperature, with a voltage of 30 V. The Chemical reagents used are from Yantai Shuangshuang Chemical Co. (Yantai, China).

The sample was sanded to 30 μm with sandpaper and then thinned using a Gatan ion thinning instrument (Gatan, Pleasanton, CA, USA), with an ion gun voltage of 6 kV. The microstructure of the ND-RD surface of the rolled specimens was characterized and observed using high-resolution transmission electron microscopy (HRTEM) with a Talos F200X field emission electron microscope (Thermo Fisher Scientific, Waltham, MA, USA), operating at an accelerating voltage of 200 kV.

## 3. Results

### 3.1. Microstructure Evolution During the Rolling Process

**Figure 2:**
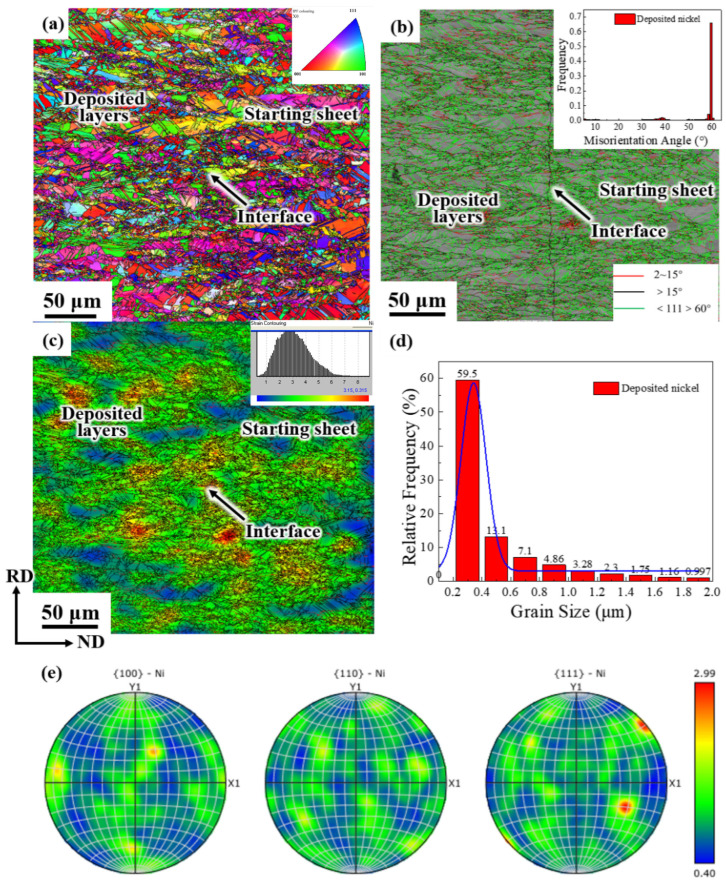
Microstructure of electrolytic nickel in the deposited state: (**a**) IPF map; (**b**) Grain boundary distribution; (**c**) Strain cloud map; (**d**) Grain size distribution; (**e**) 100, 110 and 111 pole figures from EBSD measurements.

Figure 2 presents the EBSD diagram of deposited electrolytic nickel, where the ND direction represents the deposition direction. From the grain orientation distribution shown in Figure 2a, it is evident that the grain size of the deposited electrolytic nickel is uneven, with a large number of equiaxed and columnar crystals of varying sizes. In Figure 2b, the green line represents the <111> 60° twin boundary. A large number of growth twins with varying lengths and thicknesses, accounting for over 70%, are present in the deposited electrolytic nickel. These twinning structures correspond to the lamellar structures observed in Figure 1, which either extend through the entire grain or cease growing within the grain. Pure nickel is a metal with medium to high dislocation energy, and the occurrence of numerous growth twins in electrolytic nickel has rarely been reported in the literature. Materials with high-density growth twins are generally found in electrolytic copper or other electrolytic alloys with lower dislocation energy.

Figure 2c shows the strain cloud diagram of the microstructure (strain contouring), which highlights the uneven stress distribution within the electrolytic nickel microstructure. Internal stress is generated and retained during the electrodeposition process [28]. From the grain size distribution of electrolytic nickel shown in Figure 2d, it can be observed that grains with a size of 0.3 μm account for approximately 59.5%, grains smaller than 0.5 μm make up about 72.6%, and grains smaller than 1.0 μm account for 84.56%. The average grain size of electrolytic nickel is 0.56 μm. Figure 2d shows that although the grain size distribution range of electrolytic nickel is wide, with large grains reaching over 20 μm, the overall grain size is relatively fine, with most of the grains at the nanometer level. The deposited layer near the starting sheet consists of very fine equiaxed grains, while the deposited layer farther from the starting sheet gradually exhibits columnar growth and larger grains. This phenomenon occurs because, when nickel ions first deposit on the surface of the starter electrode sheet, their nucleation rate is much higher than the crystal growth rate, resulting in the formation of a layer of fine equiaxed grains adhering to the surface. At this stage, the growth mode of electrolytic nickel grains follows a “nucleation growth mode”. During the nickel deposition process, a large number of crystal nuclei form on the cathode (the starting sheet) surface. As these crystal nuclei grow and meet, their growth halts, forming fine equiaxed crystal regions. Subsequently, when the growth rate of the crystal nuclei surpasses the nucleation rate, the crystals begin to extend along the growth direction, forming columnar crystals and transitioning into a rapid growth mode. Columnar crystals whose growth direction is parallel to the normal of the matrix are able to grow further due to sufficient space. However, the growth directions of these columnar crystals and secondary dendrites are obstructed by adjacent columnar crystals, limiting lateral growth and resulting in very small dendrite sizes. From the electrode diagram in Figure 2e, it can be seen that the texture of electrolytic nickel predominantly consists of the {220}<100> texture and <110>∥X0 wire texture.

The above research indicates that the grain size distribution range of this industrial electrolytic nickel is broad, with highly uneven grain sizes and a large number of columnar crystals and growth twins. It is a type of micro-nanocrystalline electrodeposited metal material.

**Figure 3:**
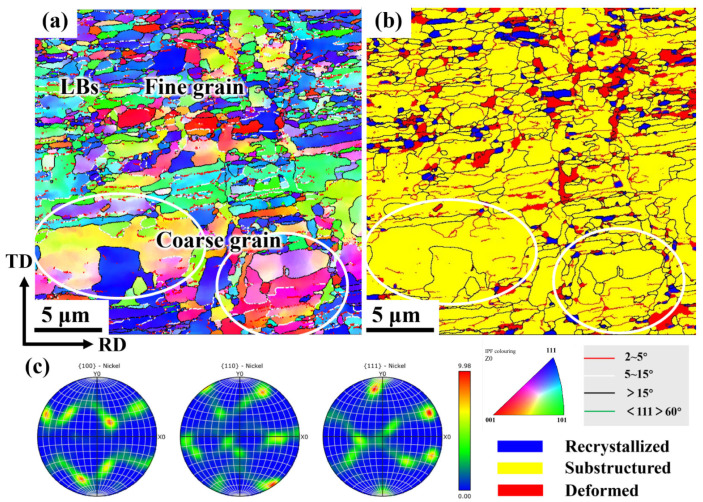
EBSD diagram of 70% CR electrolytic nickel (in the figure, the white circle indicates the recovery substructures of the coarse-grained region): (**a**) Grain orientation distribution map; (**b**) Recrystallization diagram; (**c**) 100, 110 and 111 pole figures from EBSD measurements.

To further examine the grain boundary characteristics and grain orientation distribution of electrolytic nickel during the cold deformation process, EBSD analysis was performed on the cold-rolled specimens with large deformations. Figure 3 presents the EBSD diagram of multi-channel CR 70%.

As shown in Figure 3a, the microstructure of CR 70% is divided into fine-grained and coarse-grained regions. The grains in the fine-grained region are elongated along the primary strain direction, resulting in a significant increase in the aspect ratio of the grains. A large number of lamellar boundaries (LBs) were observed during large deformation, and fibrous structures emerged after the full development of the layered organizational structure. The grain size in the coarse-grained region is approximately 5 μm or larger, with some elongated grains also distributed between the large grains.

From the recrystallization diagram (Figure 3b), it can be observed that a significant amount of recovery occurred in the microstructure of CR 70%, with a small number of recrystallized grains observed between the deformed grains and the recovery substructure. Deformed grains are more prevalent in the fine-grained region, while the coarse-grained region is predominantly composed of recovery substructures. When the CR reached 70% (Figure 3c), the grains displayed a distinct preferred orientation, primarily S texture, with a minor amount of Brass texture. Dislocation slip, which dominates plastic deformation, typically leads to lattice rotation, causing changes in the texture of the material.

**Figure 4:**
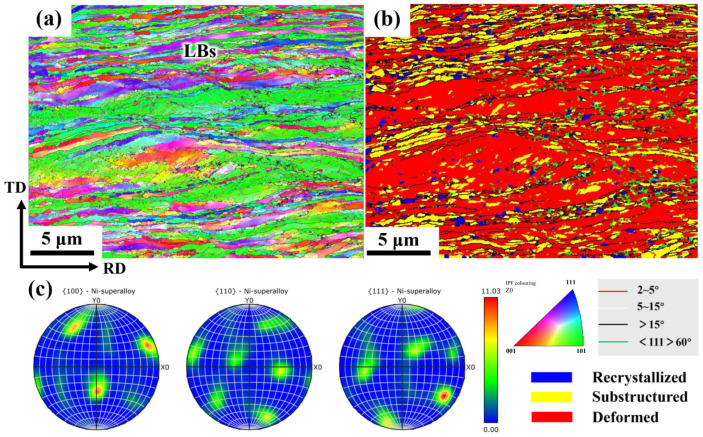
EBSD diagram of CR90% electrolytic nickel: (**a**) Grain orientation distribution map; (**b**) Recrystallization diagram; (**c**) 100, 110 and 111 pole figures from EBSD measurements.

As the deformation progressed, in the cold-rolled sample with CR 90% (Figure 4a), the grains were significantly refined and elongated, and the aspect ratio of the grains was greatly enhanced. The development of the LBs interface structure reached its peak among the microstructures. Figure 4b shows that the microstructure contains a large number of deformed structures, with some recovery structures present between the deformed areas, while recrystallized structures are sparse and scattered. With further deformation, the preferred orientation of the electrolytic nickel grains becomes more pronounced, while almost all of them transform into the S texture (Figure 4c).

**Figure 5:**
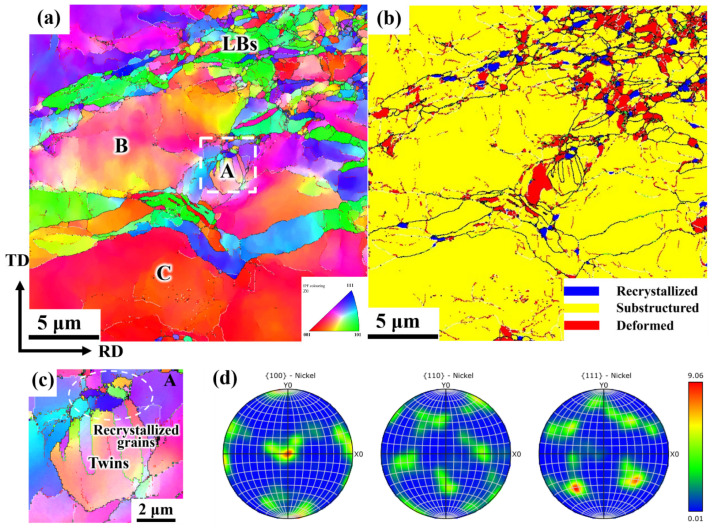
EBSD diagram of CR 98% electrolytic nickel: (**a**) Grain orientation distribution map; (**b**) Recrystallization diagram; (**c**) An enlarged view of area A in Figure 5a; (**d**) 100, 110 and 111 pole figures from EBSD measurements.

Figure 5 shows that when electrolytic nickel undergoes 98% CR deformation, it is still affected by strain and maintains the typical LBs structure under large strain, although some changes have occurred in its microstructure, with large grains exceeding 10 μm in size appearing (Figure 5a). The grains in the deformed structure region remain fine, while recovery structures have emerged in some areas (Figure 5b). During the CR process, electrolytic nickel was not annealed, but dynamic recovery occurred in certain regions of the material. This is due to the uneven deformation during the CR process, with areas of high deformation rates and large deformation amounts, resulting in adiabatic phenomena. The local temperature increased too rapidly and excessively, leading to dynamic recovery.

A close-up view of area A in Figure 5a (Figure 5c) reveals the formation of an equiaxed grain structure, within grains enclosed by <111> 60° grain boundaries, indicating the presence of twins. During the cold deformation process, with a deformation degree as high as 98%, all grains are affected by large strain, becoming flattened, elongated, and refined. The appearance of equiaxed crystals at this stage suggests that some uncommon phenomena have occurred in these regions, such as recovery recrystallization due to thermal effects. Similar occurrences have been reported in pure nickel subjected to high-pressure torsional (HPT) deformation [18]. In electrolytic nickel with 98% CR, deformable textures dominated by Brass and S, as well as a small amount of recrystallized textures, including Cube and R, were observed (Figure 5d).

### 3.2. Grain Boundary Character Distribution

**Figure 6:**
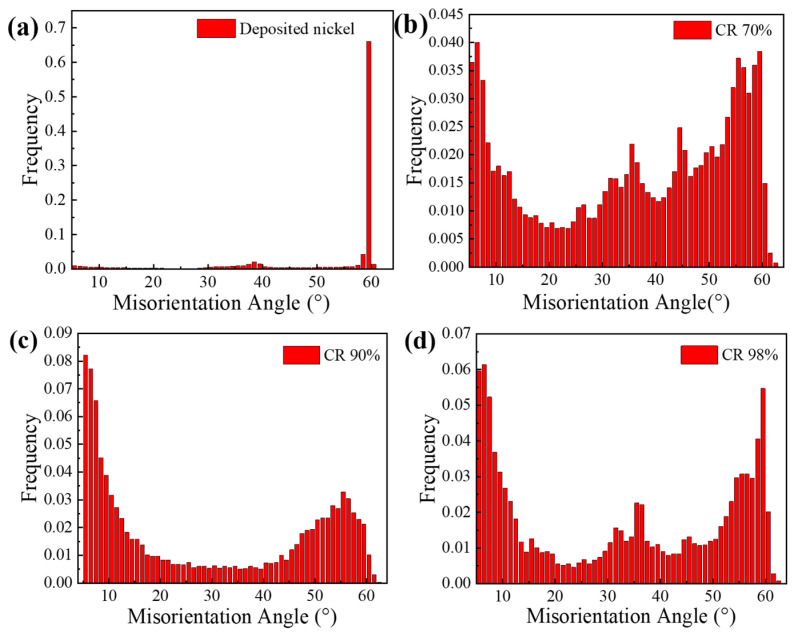
Distribution map of orientation difference of electrolytic nickel during cold rolling process: (**a**) Sedimentary state; (**b**) CR 70%; (**c**) CR 90%; (**d**) CR 98%.

Figure 6 illustrates the distribution of orientation differences in electrolytic nickel after varying degrees of large strain. Figure 6a shows the grain orientation difference distribution of the deposited electrolytic nickel. It is evident that a large number of grains dominate at an angle of 60°, which corresponds to the growth twin boundary angle. During the deformation process, the formation of S-bands and shear bands can cause adjacent crystals to rotate. In the case of large deformations, interface merging also leads to an increase in orientation differences. As CR deformation proceeds, the grains near the grain boundaries at 60° decrease significantly, and consequently, large quantities of LAGBs (Low angle grain boundaries) and HAGBs (High angle grain boundaries) appear (Figure 6b–d). During large deformation, the grains rotate to different preferred orientations, thereby forming a strong deformed texture. The components of different textures may deviate significantly, resulting in a substantial difference in interface orientation. When CR reaches 90% (Figure 6c), the distribution of grain boundary orientation differences shows polarization. At this stage, the proportion of LAGBs is highest, and a large number of small-angle grain boundaries emerge, contributing to an increase in material strength.

**Figure 7:**
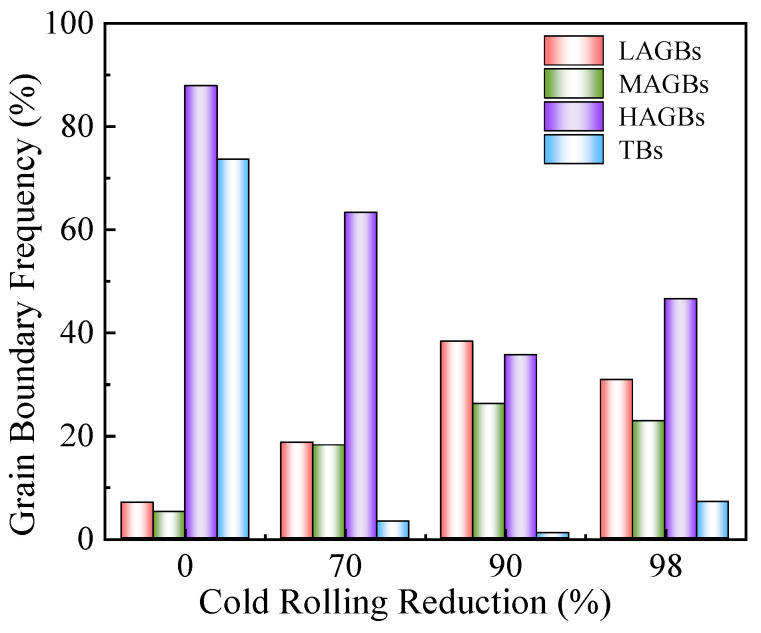
Grain boundary statistics during cold rolling of electrolytic nickel (in the figure, LAGBs represents the 2–5° grain boundary, MAGBs represents the 5–15° grain boundary, HAGBs represents the > 15° grain boundary, and TBs represents the twin boundary).

Figure 7 presents a statistical chart of the changes in the proportion of grain boundaries in electrolytic nickel during the CR process. As deformation progresses, the proportions of HAGBs and TBs (Twin boundaries) decrease significantly, while the proportions of LAGBs and MAGBs (Middle angle grain boundaries) increase markedly. The increase in these dependent variables causes dislocations to continuously accumulate at the grain boundaries, leading to the division and movement of the grain boundaries. This results in the rotation of large-angle grain boundaries at smaller angles and the appearance of numerous substructures. At this point, the structure becomes highly unstable.

### 3.3. Microstructure Evolution of Cold Rolling and the Regression Mechanism of Growth Twins of Electrolytic Nickel Under Small Deformation

**Figure 8:**
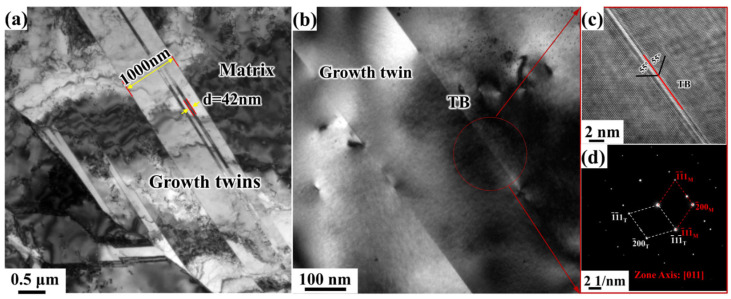
TEM microstructure observation of electrolytic nickel: (**a**) Bright-field image of the deposited state; (**b**) Growth twinning bright-field image; (**c**) HRTEM image of the twin boundary; (**d**) is the diffraction pattern of the twin boundary in the area delineated by the ring in (**b**).

Figure 8 shows the TEM (Transmission electron microscope) morphology of the deposited electrolytic nickel sample. As seen in Figure 8a, the grain size is highly nonuniform and contains numerous randomly oriented lamellar structures. The HRTEM image of the grain boundaries in Figure 8b and its magnified view in Figure 8c, along with the corresponding selected area electron diffraction (SAED) pattern in (Figure 8d, confirm that the grain boundaries of the lamellar structure are CTBs (Coherent twin boundaries), with a few dislocations observed within the crystal matrix. Additionally, the distribution of the twinned layers is uneven and varies significantly in size. Specifically, the maximum twin length exceeds 20 μm, while the thickness of the layers ranges from several tens of nanometers to approximately 1000 nm.

For metals with medium to high stacking fault energies, the formation of growth twins is typically difficult. In most pure nickel systems, twinning generally occurs through annealing processes. Previous studies have shown that when thick films are fabricated by magnetron sputtering, both pure and Al-doped nickel can exhibit nanoscale TBs [21,29,30]. The occurrence of a high density of growth twins in electrodeposited pure nickel is largely attributed to the presence of impurity elements in the electrolytic nickel [31,32]. This industrial-grade electrolytic nickel exhibits a wide grain size distribution, with the coexistence of micro- and nano-sized grains and a high density of growth twins. The microstructure of this bulk electrodeposited metal is thus highly complex, which will have a profound and multifaceted impact on its plastic deformation behavior.

**Figure 9:**
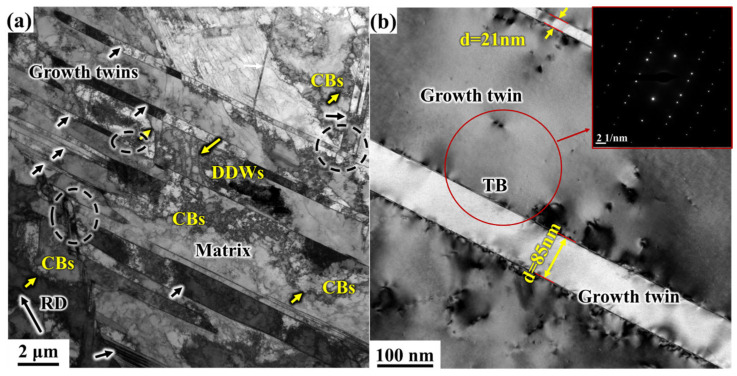
TEM microstructure observation of 5% electrolytic nickel CR: (**a**) Bright-field image; (**b**) Growth twinned bright-field images and diffraction patterns of TB.

Figure 9 illustrates the transmission electron microstructure of the electrolytic nickel sample after CR 5%. At this stage, although the deformation is minimal, meaning that the electrolytic nickel has only just begun to experience deformation, dislocations can be observed to appear rapidly within its internal structure (Figure 9a). These dislocations accumulate and settle into dislocation interfaces during the plastic deformation process, subdividing the grains into cells and CBs with different structures [33]. At low strain, long, and straight dislocation interfaces (DDWs, Density dislocation walls) gradually form, segmenting the grains into different CBs (Cell blocks), which help accommodate the strain of adjacent cell blocks. Geometrically necessary boundaries (GNBs) [20,34] are also formed between the CBs. These interfaces arise from the interaction of dislocations in different slip systems, facilitating the coordination of inhomogeneous deformation.

At this stage, the growth twin boundaries that formed during the nickel electrodeposition process remain clearly visible (Figure 9b), and the corresponding SAED patterns indicate that the twin orientation relationship has not changed. It is observed that in the figure, one twinned layer has a thickness of 21 nm, while another has a thickness of 85 nm.

During the CR process, the grains within electrolytic nickel are gradually elongated due to the rolling deformation, and local areas begin to deform. These areas are also affected by strain, causing the torsion angles of grains with different orientations to vary. Due to the anisotropy of plastic deformation, dislocations preferentially propagate along the twin boundaries [35]. Dislocations on TBs and within the twins begin to proliferate rapidly. Twinned lamellae aligned with the rolling strain direction experience minimal change, while those at 45° or other orientations are more affected. Some twin layers with different directions begin to interact, as indicated by the circular area in Figure 9a.

**Figure 10:**
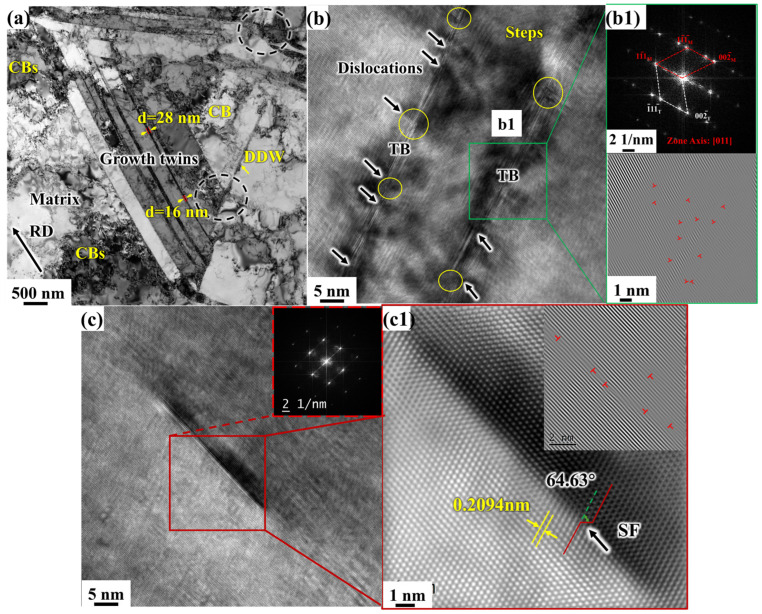
TEM microstructure observation of CR 10% electrolytic nickel: (**a**) Bright-field image; (**b**) The high-resolution image of the twin boundary and (**b1**) showing diffraction spot patterns and Fourier inversion transformations; (**c**) The diffraction pattern of the stratification fault; (**c1**) high-resolution image and Fourier inversion image.

Figure 10 presents the TEM microstructure of the 10% CR sample. Although the deformation at 10% is small, the TBs are inevitably affected by dislocation movement, and dislocations are emitted along the TBs. As shown in Figure 10a, with increased deformation, dislocations in the internal structure continue to proliferate, and the formation rate of dislocation cells accelerates. More growth twins squeeze the surrounding matrix structure, and further cuttings occurs between the twinned lamellae (indicated by the circular area in Figure 10a). Growth twins in different directions are affected by strain, leading to elongation and bending. Twins along the direction of maximum shear stress exhibit significant bending, while those parallel to the rolling direction become elongated and thinner. The thickness of the same twinned layer changes from 28 nm to 16 nm, as twinning is influenced by deformation, resulting in an increase in length and a decrease in thickness (Figure 10a).

The originally straight TBs gradually exhibit evolutionary characteristics as deformation progresses. The TBs undergone significant shear deformation. In Figure 10b, a large number of dislocations accumulate along the TBs. TBs that are perpendicular or inclined at a certain angle to the strain dislocate more rapidly, resulting in the formation of multiple steps. These steps progressively divide the grain boundaries until they are sheared off. Subsequently, the TBs that were cut off break into sections of interfaces (Figure 10b). However, due to the small amount of deformation at this stage, most areas still maintain a well-defined twinned lamellar structure, and the corresponding SAED pattern (Figure 10b1) indicates that the twin relationship has not changed.

As the deformation progresses, more dislocations become entangled into clusters, leading to a dense packing of dislocations. The sawtooth pattern of the TBs intensifies, eventually blurring the growth twin boundaries. Dislocations produced and emitted by the TBs gradually expand into the crystal matrix, forming numerous dislocation cells within the twins. Stacking faults (SFs) were observed in the deformed microstructure of electrolytic nickel at small strains, as shown in Figure 10c. Similar to the twinning phenomenon in pure nickel, delamination is less likely to occur. At this stage, the occurrence of layer faults may be linked to the high number of growth twins formed during electrodeposition.

**Figure 11:**
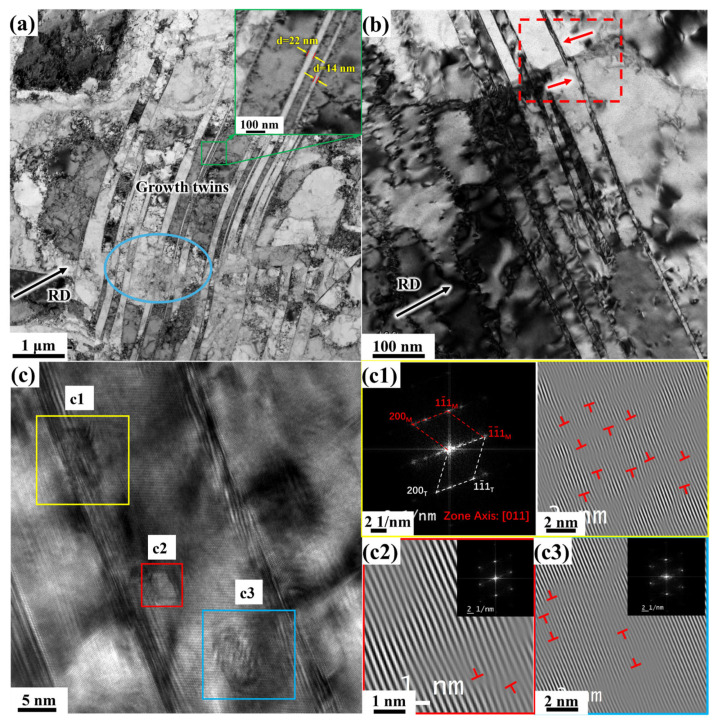
TEM microstructure observation of 30% CR electrolytic nickel: (**a**) Bright-field image; (**b**) Twins with multiple twin boundaries blocked and broken by dislocations; (**c**) HRTEM images of the twin boundaries, as well as the diffraction patterns and Fourier inversion images in different regions: The grain boundary at (**c1**) still maintains a twin relationship, while (**c2**,**c3**), as indicated by the diffraction patterns, are the matrix materials.

Figure 11 shows the microstructure of the 30% CR sample. As shown in Figure 11a, with increasing strain, the twinned layers continue to elongate, and their curvature becomes more pronounced. One twin is observed to be 22 nm thick, while another is only 14 nm thick, indicating that the growth twins are continuously elongated and thinned under strain. A large number of dislocations accumulate near the TBs, which differs from the initial state of straight, clean growth twin boundaries before deformation. Large twinned lamellae can accommodate shear strain through intragastric dislocation motion, while fine twinned lamellae, due to the size limitation of dislocation movement, cannot coordinate shear strain through dislocation motion alone. Instead, they accommodate shear strain through dislocations and TBs reaction-induced detwinning. A large number of dislocations interacting with the TBs induce continuous dislocation reactions, causing a significant proliferation of dislocations along the TBs and their accumulation, resulting in twin bending. At the shear strain concentration point, the growth twins in the electrolytic nickel have started to fade, as shown in the circular area of Figure 11a. Multiple twinned lamellae become difficult to distinguish, and the twin boundaries are completely blurred.

As shown in Figure 11b, a large number of dislocations are entangled within the growth-twinned lamellae. Under the influence of local strain, dislocations accumulate in a direction perpendicular to the twin boundaries, causing multiple twins to collectively deflect. Due to the deformation, the twinned lamellae not only bent but also experience breakage. A twinned lamella completely disconnected by dislocation cutting was observed (at the position marked by the short dashed box in Figure 11b).

Figure 11c shows the characterization of the twinned microstructure by HRTEM. The diffraction pattern corresponding to the c1 grain boundary in the figure still maintains the twin relationship, though it slightly deviates from the original crystal band axis. This indicates that only a small deflection has occurred in the orientation between the twin and the matrix. On these incompletely dissipated twins, dislocations continue to be emitted from the TBs, proliferating and moving near the twin boundaries until they accumulate and entangle. The dislocation density near the TBs increases progressively. As strain increases, new slip systems are activated to coordinate the deformation. During the continuous movement of dislocations, the TBs can no longer impede dislocation motion, resulting in a large number of movable incomplete dislocations on the TBs. Under the mutual interaction of dislocations and TBs, the originally continuous and straight TBs become discontinuous and eventually break under continuous strain. The original growth twins in electrolytic nickel soon disappear.

**Figure 12:**
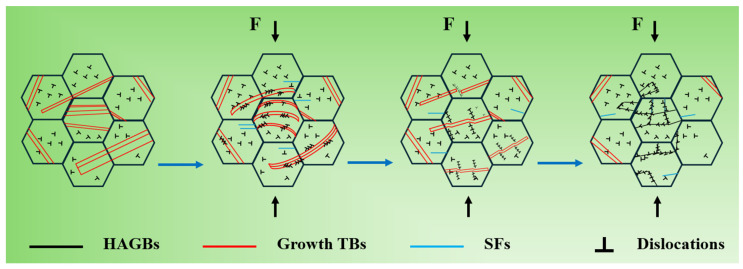
Schematic diagram of detwinning mechanism in the cold rolling process of electrolytic nickel.

Under dislocation motion, different regions of the twins undergo various changes. The unique mechanical behavior of twins stems from their inherent deformation mechanisms. Research [36,37] indicates that when an edge dislocation encounters Σ3 TBs, the interaction between the dislocation and the TBs generates a new edge dislocation, which can slip within the twinned lamellae. Simultaneously, a new incomplete dislocation is created at the TBs, which can also slide along the TBs. In this manner, the original growth-twinned lamellae in electrolytic nickel not only impede dislocation motion but also act as slip planes for dislocations, absorbing and storing dislocations during deformation. This mechanism strengthens the metallic material while improving its macroscopic plasticity [38]. Thus, under CR conditions, during the initial stage of deformation of electrolytic nickel, the plastic deformation is predominantly governed by the detwinning mechanism, which involves extensive interaction between dislocations and twins. A schematic diagram of this mechanism is shown in Figure 12.

### 3.4. Evolution of Cold-Rolled Microstructure and Cold Plastic Deformation Mechanism Under Large Deformation

**Figure 13:**
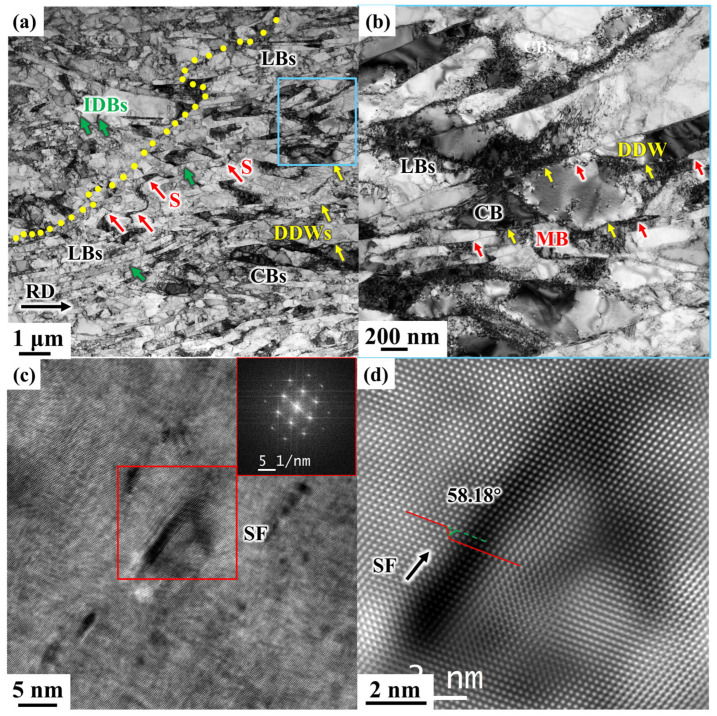
TEM microstructure observation of 70% electrolytic nickel CR: (**a**) Bright-field image; (**b**) Enlarged view of the box in (**a**); (**c**) Laminar faults and their diffraction patterns; (**d**) HRTEM image of the middle layer error in (**c**).

After 70% deformation in CR, the deformed structure is shown in Figure 13. As shown in Figure 13a, with increasing deformation, the number of dislocation cells increases, the grains become progressively finer, and dislocation proliferation continuously enhances the strength of the material. These fine grains are formed through the evolution of dislocation structures and the shearing and fragmentation of growth twins. S-bands are also observed (indicated by the red arrows in Figure 13a), which from when intense shear occurs due to local large shear strains. At 70% CR, the deformation degree transitions from medium strain to large strain, and at this point, the microstructure contains both typical small-strain and large-strain structures, making the deformation structures in the CR 70% microstructure highly diverse.

During plastic deformation of metals, different CBs form within the grains, and slight orientation differences between these cell blocks result in variations in the slip systems activated by each block. The interface structure of DDWs/MBs (Microbands)/LBs forms GNBs, while the boundaries of dislocation cells and the interfaces between layered interfaces are classified as IDBs (Incidental dislocation boundaries), as indicated by the green arrow in Figure 13a). IDBs are dispersed interfaces located between GNBs and retain distinct dislocation characteristics. The interaction between dislocations within the cell blocks constantly generates new IDBs as strain increases from small to large.

The lamellar structure, often referred to as LBs, gradually forms when metals undergo large strains, as shown in Figure 13a. This structure is composed of DDWs and CBs, and is a characteristic feature of medium- to high-energy metals under significant deformation [20]. Each layer contains several cell blocks. As stress increases, individual DDWs gradually widen and start to split into two or more DDWs. The splitting of original DDWs is the most common mechanism for the formation of new CBs. After splitting, DDWs exhibit strip-shaped morphological characteristics under a transmission electron microscope, which are known as MBs, as shown in Figure 13b.

FCC metals have multiple slip systems, but pure nickel is not prone to twin deformation during plastic deformation. Only under low-temperature and high-strain rate conditions, when dislocation motion is hindered, can a deformed twin structure form [35]. After 70% CR deformation, it is difficult to observe the presence of twin boundaries in the microstructure of electrolytic nickel. The growth twins essentially disappear under the influence of large deformation. Consequently, the electrolytic nickel, which initially contained a large number of growth twins, not only sees the disappearance of growth twin post-deformation but also shows minimal appearance of deformation twins. Under 70% large strain, a significant number of laminar structures appear in the electrolytic nickel microstructure (Figure 13c). During the cold deformation process of electrolytic nickel, the deformation mechanism is characterized by the coexistence of dislocation slip and dislocation interaction.

**Figure 14:**
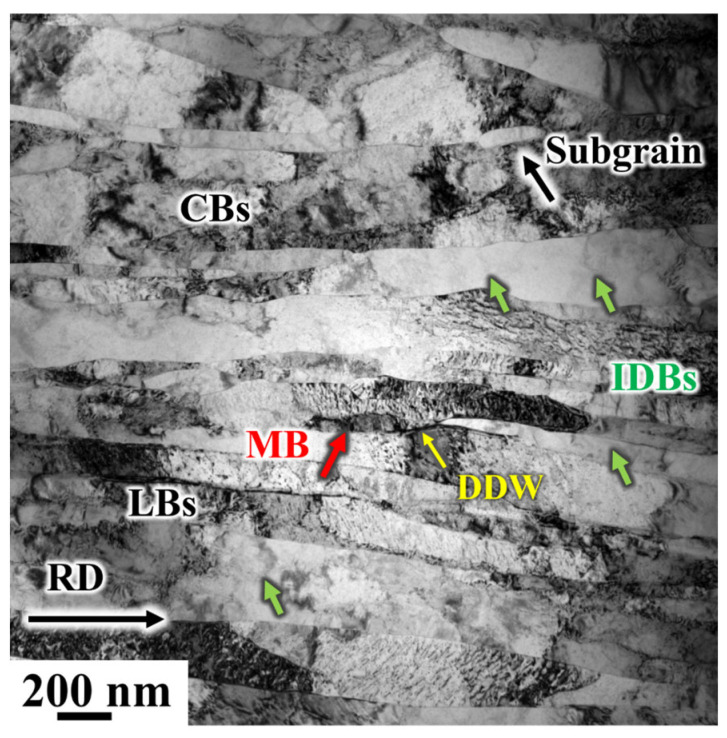
TEM microstructure observation of 90% electrolytic nickel CR.

After 70% deformation by CR, the microstructure transitions from MBs/DDWs inclined towards the rolling plane to LBs nearly parallel to the rolling direction. Figure 14 shows the microstructure of the CR 90% sample, where the microstructure has almost entirely evolved into LBs. The width of these LBs ranges from 200 nm to 600 nm. The lamellar structure is not perfectly straight but gradually bends along with local structures or local shear zones.

Under moderate strain, a new dominant slip system rapidly emerges, transforming the structure from one associated with small strain to one characteristic of large strain [39]. The spacing between IDBs and GNBs continuously decreases as strain increases. The thinned layers continue to deform and are further subdivided by IDBs into the typical high-strain structure (Figure 14). Inside the LBs, a large number of entangled dislocations and CBs are observed, while between the layers, a few DDWs and MBs are present.

Under the influence of large strain, due to the movement of dislocations and the interaction between dislocations and laminates, the original CBs gradually elongate and refine into smaller CBs. Simultaneously, the number of MBs increases, and the orientation difference between the matrix and MBs also becomes more pronounced. Ultimately, as cumulative deformation progresses, the MBs further refine and eventually disappear, leading to the formation of subcrystals.

**Figure 15:**
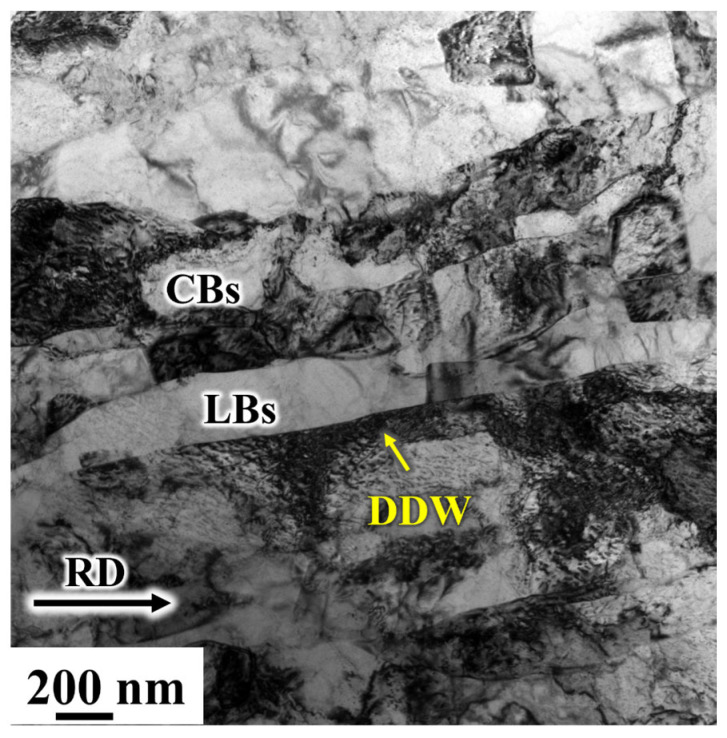
Observation of the morphology and microstructure of 98% CR electrolytic nickel.

Figure 15 shows the transmission electron microstructure of the sample at CR 98%. The LBs lamellar structure is still visible in the figure, which is thicker compared to the lamellar structure at 90% CR. CBs are also present. With increasing deformation, at CR 98%, the interfacial spacing between the layers did not continue to decrease as observed in polycrystalline nickel at CR 98% [20], nor did it form large-angle grain boundaries under the combined effects of structural and texture evolution [40]. Instead, the spacing between the layers increased.

### 3.5. Grain Size Distribution

**Figure 16:**
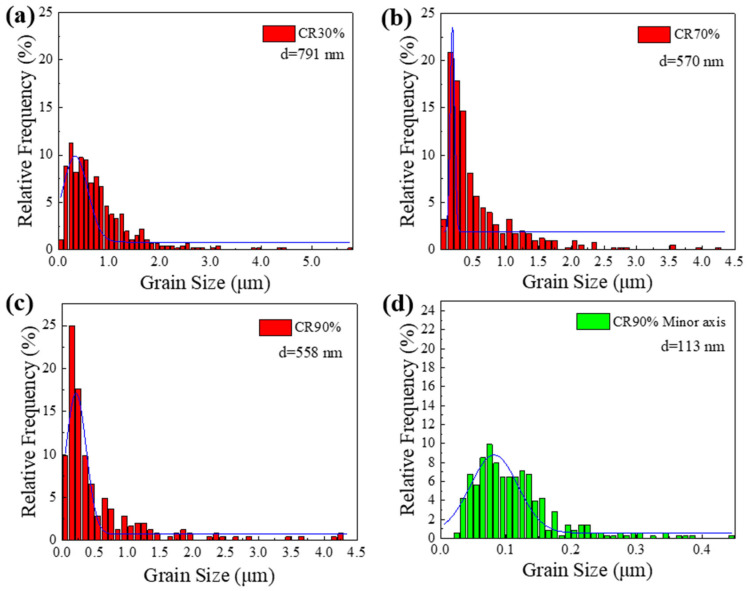
Distribution of electrolytic nickel grain size during cold rolling process: (**a**) CR 30%; (**b**) CR 70%; (**c**) CR 90%; (**d**) Short axis direction of CR 90% lamellar grains.

The changes in the size distribution of electrolytic nickel grains during the CR 30%, CR 70%, and CR 90% deformation processes were statistically analyzed using TEM images, as shown in Figure 16. A significant grain refinement was observed throughout the cold plastic deformation process.

It should be noted that, based on the previous analysis, a large number of substructures appear at CR 98%, which affects the accuracy of grain distribution observations. Therefore, no statistics were conducted for this deformation level. The average grain size at CR 30% was 791 nm. As strain increased, the average grain size decreased to 570 nm after CR 70%. Following CR 90%, a large number of lamellar structures emerged. The lamellar thickness, or the size in the short-axis direction of the grains, was statistically analyzed. After CR reached 90%, the average short-axis grain size was refined to 113 nm, representing the refinement limit for electrolytic nickel during CR. At this point, the degree of work hardening is maximal, and the annihilation and proliferation of dislocations reach equilibrium.

### 3.6. Microhardness of Electrolytic Nickel at Different Pressing Amounts

**Figure 17:**
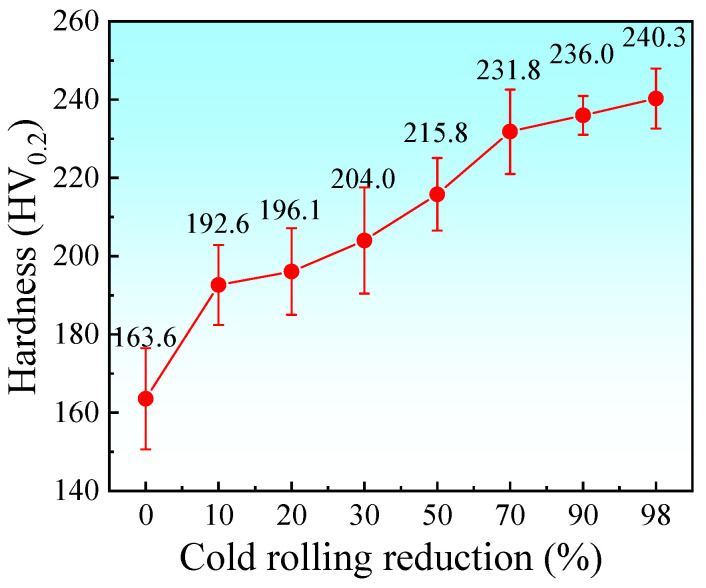
Microhardness of cold-rolled electrolytic nickel with different reduction amounts.

Figure 17 presents the microhardness values of electrolytic nickel after CR with varying degrees of deformation. As the deformation degree increases, the hardness of the electrolytic nickel rises significantly. The average microhardness of the deposited electrolytic nickel is 163.6 HV. After 10% CR, the average microhardness increases from 163.6 HV to over 192 HV. At 30% deformation, the microhardness value reaches 204.0 HV. With a deformation degree of 50%, the average microhardness is 215.8 HV, and the material continues to strengthen. When CR reaches 70%, the average microhardness is 231.8 HV, representing a cumulative increase of more than 41%, with the strengthening rate remaining relatively high.

As deformation continues, the rate of hardness increase begins to decline, and the hardness value shows only a slight rise. This indicates that the microstructure of electrolytic nickel tends toward a relatively stable state with increasing strain. When the CR reduction reaches 98%, the average microhardness attains the maximum value during the entire deformation process, reaching 240.3 HV. Compared with the undeformed state, this corresponds to a cumulative increase of 46.88% in microhardness.

During the cold-rolling-induced strain hardening of electrolytic nickel, the primary microscopic mechanism of strengthening is the accumulation of dislocations at grain boundaries. The resistance to dislocation slip is associated with the interfacial misorientation of small-angle grain boundaries. A larger misorientation results in more significant obstruction to dislocation motion, with a strengthening effect comparable to that of large-angle grain boundaries [41]. Previous studies [42] have shown that the critical misorientation angle typically ranges from 2° to 5°. Therefore, as the degree of rolling deformation increases, the proportion of small-angle grain boundaries continuously rises, which is considered the main factor contributing to the rapid increase in the hardness of electrolytic nickel.

## 4. Discussion

### 4.1. Dynamic Recovery and Recrystallization During the Cold Rolling Process

**Figure 18:**
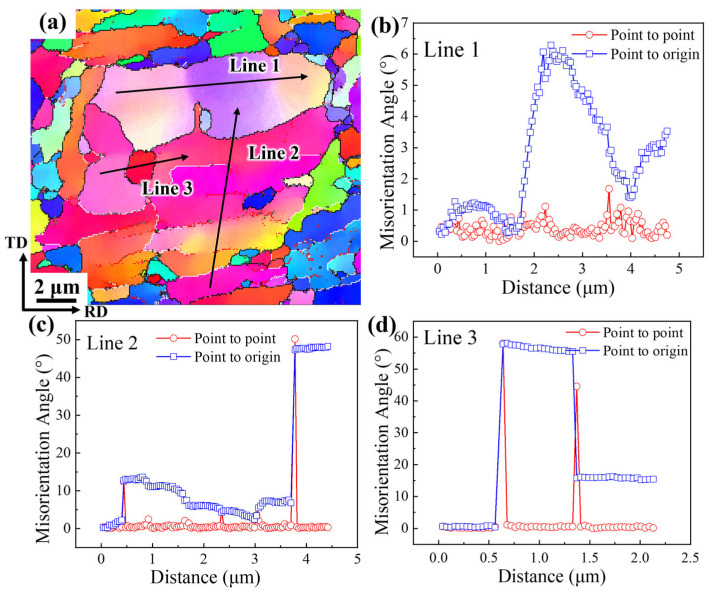
Recovery and recrystallization in CR 70%: (**a**) Grain orientation distribution map; (**b**) Original orientation difference maps of Line 1 point-to-point and point-to-point; (**c**) Original orientation difference maps of Line 2 point-to-point and point-to-point; (**d**) Original orientation difference maps of Line 3 point-to-point and point-to-point.

Further analysis reveals the occurrence of recovery and recrystallization phenomena in the microstructure at 70% CR, as shown in Figure 18. Figure 18a displays the EBSD scan along Line 1, and Figure 18b illustrates the corresponding point-to-point and point-to-original orientation differences. The cumulative misorientation within this region reaches approximately 6°, indicating relatively minor internal orientation variation. This suggests that several adjacent subgrains have coalesced during the recovery process, accompanied by the reappearance of subgrain boundaries.

For Line 2, the point-to-point and point-to-original orientation differences are shown in Figure 18c. This area is still undergoing recovery, with subgrain boundaries that have not fully subsided. The cumulative orientation difference exceeds 13.62°, indicating a relatively high misorientation that facilitates grain boundary migration. The grains along both Line 1 and Line 2 exhibit high-angle grain boundaries, with a misorientation angle of 50.17°, further confirming the presence of recovery in these regions.

Figure 18d shows the orientation analysis of the region along Line 3, with measured grain boundary misorientations of 58.08° and 44.05°, respectively. These values indicate that the grain is enclosed by large-angle grain boundaries and has likely formed as a new grain following boundary migration during recrystallization. Therefore, the region illustrated in Figure 18 is identified as having undergone thermally activated processes, including both recovery and recrystallization.

**Figure 19:**
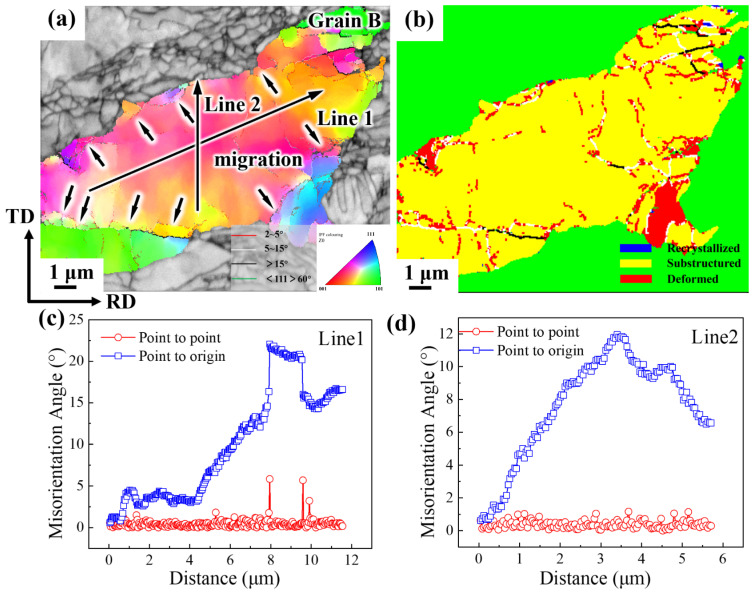
Orientation analysis of grain B in CR 98%: (**a**) Distribution map of grain orientation; (**b**) Recrystallization diagram; (**c**) Original orientation difference maps of Line 1 point-to-point and point-to-point; (**d**) Original orientation difference maps of Line 2 point-to-point and point-to-point.

Figure 19 analyzes the orientation of grain B in the CR 98% condition (Figure 5). As shown in Figure 19a, the size of grain B exceeds 10 μm, which is several dozen times larger than the grain size of 200 nm to 400 nm observed in the 90% condition. Near grain B, a large-strain LBs structure is also observed. A significant number of fine grains are distributed within the LBs, and both small-angle grain boundaries and a large concentration of dislocations are present in this region. When a large grain appears within the microstructure, its orientation difference is not only substantial but its grain boundaries transform into large-angle boundaries, indicating that this grain has experienced sudden growth, as shown in Figure 19b.

Within this grain, there are few dislocations, and almost no internal substructures are observed. At the grain boundary edges, subgrain boundaries and subgrains, delineated by small-angle (2–5°) and medium-angle (5–15°) grain boundaries, are distributed, as shown in Figure 19a. Originally, a high-strain structure should contain a large number of subgrain boundaries; however, the number of subgrain boundaries within grain B has decreased, and the remaining subgrain boundaries are observed to migrate in all directions.

The distribution of orientation differences along Line 1 and Line 2 within the grains was analyzed (Figure 19c,d). The cumulative orientation difference in the Line 1 direction exceeded 20°, while in the Line 2 direction, it reached only 12°. In the figure, the direction of Line 1 is aligned with the primary strain direction of rolling, which is mainly influenced by deformation mechanisms such as elongation and torsion. The Line 2 direction, being perpendicular to the main strain direction, is more affected by compressive deformation. As a result, the cumulative orientation differences vary between the two directions within the same grain. During the deformation process, the original grains elongate along the rolling direction and twist along a specific axial direction, forming numerous new subgrain boundaries GNBs composed of geometrically necessary dislocations in the microstructure. Affected by local deformation heating, these subgrain boundaries rapidly migrate and merge, ultimately forming large-angle grain boundaries.

In the study of the effect of reduction on the CR process of strip steel [43], it was found that the reduction has a significant impact on temperature. The average temperature at the outlet of the strip can reach up to 268 °C, and the transverse temperature distribution is also uneven [44]. It can be inferred that when electrolytic nickel is cold-rolled to 98%, its accumulated strain and deformation energy are quite high, with local strains exceeding the average strain of the material. Under these conditions, adiabatic temperature rise may occur in localized regions during the CR of electrolytic nickel [45]. After the adiabatic temperature rise, heat exchange in these regions becomes difficult. Under the influence of thermal activation, the microstructure in these areas undergoes rapid dynamic recrystallization, followed by grain growth, while other regions retain the deformation characteristics of large strain. This recrystallization occurs during the CR deformation process, but its mechanism is similar to dynamic recrystallization in hot deformation. However, during the CR process, only a few areas are affected by the adiabatic temperature rise, with most areas still exhibiting the microstructural features typical of large cold plastic deformation.

**Figure 20:**
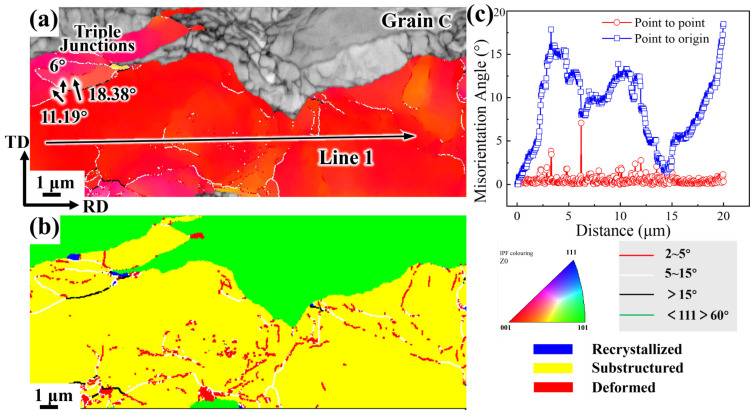
Grain C orientation Analysis in CR 98%: (**a**) Grain orientation distribution map; (**b**) Recrystallization diagram; (**c**) The original orientation difference diagrams of point-to-point and point-to-point within the grains.

Figure 20 analyzes the orientation of grain C in the CR 98% condition (see Figure 5). The behavior of grain C is similar to that of grain B in Figure 19. The size of grain C exceeds 10 μm, and the grains has also experienced sudden growth (Figure 20a). Some substructures and low-angle grain boundaries, below 15°, remain within the grains (Figure 20b). In the point-to-point and point-to-original orientation difference analysis shown in Figure 20c, the cumulative orientation difference within the grain exceeds 17°. The relatively large misorientation is also formed by the coalescence of several subgrains, indicating that the formation of large grains is highly likely the result of the rotation and merging of small-angle grain boundaries. During the rotation and merging of subgrains into large grains, small-angle grain boundaries disappear, suggesting that local recovery and recrystallization have occurred, though the degree of microstructural recrystallization is not high.

Research has shown that after HPT ultra-high strain, isoaxial crystals are formed within the pure nickel microstructure [18,46]. This may be due to the fact that during the structural evolution process, the lamellar structure LBs undergoes greater strain, leading to structural reorganization aimed at reducing interfacial energy. However, during the CR deformation process, the cumulative deformation reaches 98% (with a strain of 4.5), which is much smaller than the HPT strain of 12. Therefore, the appearance of large grains in the microstructure in this case cannot be attributed to a structural reorganization to reduce interfacial energy.

During the large deformation of electrolytic nickel in CR, non-uniform deformation results in a high local cumulative strain. Affected by the heat generated during the rolling deformation, the temperature in regions with high strain will be further elevated. In these high-strain regions, the grains are also finer, the grain boundary energy is higher, and the structure becomes very unstable, providing a kinetic basis for recovery and recrystallization. From a thermodynamic perspective, cold-deformed metals are inherently unstable. When appropriate dynamic conditions exist, they will transform into a low-energy state, eliminating deformation-induced damage and restoring the microstructure to its pre-deformation state. During the large deformation of electrolytic nickel, local heating causes a temperature rise, which effectively initiates a thermal deformation process. This leads to rapid dynamic recovery and recrystallization. Additionally, due to the high local temperature, the grains grow rapidly after recrystallization.

### 4.2. Twins Formed During the Cold Rolling Process

**Figure 21:**
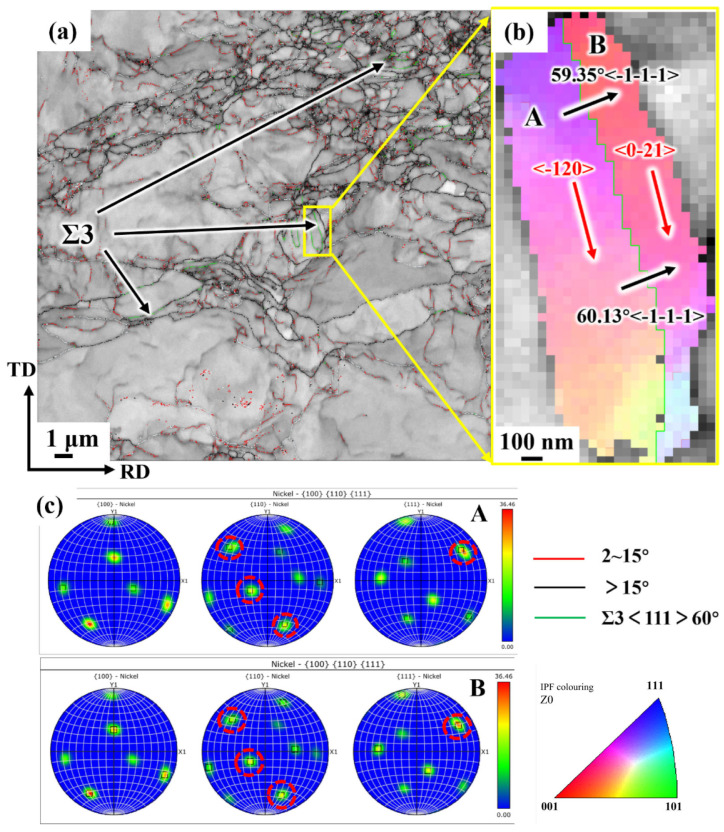
Twins of electrolytic nickel during cold rolling: (**a**) Grain boundary diagram of CR 98%; (**b**) Twin boundaries and the orientation of the two twin relationship grains; (**c**) 100, 110 and 111 pole figures from EBSD measurements of twins A and B.

Figure 21 characterizes the twinned structure observed in the CR 98% sample. Figure 21a presents the typical grain boundary structure of a sample subjected to a cumulative CR deformation of 98%. It can be observed that the deformed structure consists of elongated grains, equiaxed grains, and large grains of varying sizes. A significant proportion (>7%) of the grain boundaries between these grains are 60° overlapping position lattice CSL Σ3 grain boundaries.

To further investigate the grain boundary structure, grains A and B are marked in Figure 21b, and their pole figures for {110} and {111} are shown in Figure 21c. It is evident that the two grains share three sets of {110} poles and one set of {111} poles, indicating that these grains have a twin orientation relationship, with the grain boundary between them being a twin boundary.

In Figure 21b, the grain directions of grain A and grain B are also indicated by arrows. The grain boundary between the two grains is not parallel to any {111} grain direction, which suggests that it is not a coherent {111} twin boundary. By comparing the grain boundary direction with possible crystal plane indices, it was found that the Σ3 grain boundary in grains A and B is closely parallel to the <−20> and <0–21> directions, respectively, indicating that the grain boundary plane lies on the {120} crystal plane.

Under most conditions, except for extreme cases such as extremely high deformation strain rates, it is difficult to observe the occurrence of twins during the deformation of coarse-grained pure nickel. In this study, a small number of Σ3 twins were observed during the CR process of electrolytic nickel. Examination of the Σ3 grain boundaries reveals that they are Σ3 {120} type incoherent twin boundaries (ITBs). The reason for this phenomenon is that there is deformation inhomogeneity during the large deformation process, and the strain concentration in the local area of the material will accumulate a large amount of deformation heat energy in a short period of time, which cannot be dissipated, resulting in a rapid rise in temperature. This adiabatic effect induces annealing twins in the local area of the material. These findings provide strong evidence for the formation of noncoherent twins during the deformation of metallic materials with mid-to-high fault energy.

Σ3 {111} coherent twin boundaries CTB exhibit a strong obstructive effect on dislocation slip and can effectively enhance the material strength [47]. Additionally, CTB can absorb and transmit dislocations, thereby contributing to the macroscopic plasticity of metals. Although the grain boundary strength of Σ3 {111} ITB is lower than that of CTBs, their similar stable properties allow ITBs to still improve the performance of the material.

## 5. Conclusions

This paper investigates the rolling deformation of electrolytic nickel at room temperature and characterizes and analyzes the microstructural features at various deformation degrees ranging from 5% to 98%. The intrinsic microstructure of electrolytic nickel is complex, with a high density of growth twins. During the room-temperature rolling deformation process, the growth of twinned sheets not only hinders dislocation movement, thus strengthening the metal, but also serves as a slip plane for dislocations, absorbing and storing dislocations during deformation and enhancing macroscopic plasticity. Under the continuous interaction between dislocations and growth twin boundaries, these boundaries can dissipate at medium and low deformation degrees, thereby reducing local shear strain during coordinated cold plastic deformation.

The evolution of the deformation structure with strain is as follows: at medium and low deformation degrees (up to 30%), dislocation cells and extended dislocation boundaries are predominantly formed. The interaction between dislocations and twins is the main deformation mechanism, and the detwinning mechanism plays a key role in coordinating local shear strain. When the CR reaches 70%, a typical bamon-like LBs structure begins to appear, accompanied by a large number of dislocations in the microstructure. During the large deformation of electrolytic nickel, a deformation mechanism where dislocation slip and dislocations coexist occurs simultaneously. When CR reaches 90%, the microstructure has almost entirely evolved into LBs, with the average short-axis size refined to 113 nm, which marks the refinement limit for electrolytic nickel CR.

When CR is deformed to 70% and 98%, dynamic recovery and recrystallization phenomena occur in the electrolytic nickel. During the large deformation process, due to the high accumulation of strain, local strain becomes even higher. The deformation heat generated leads to local heating, resulting in “dynamic recovery and dynamic recrystallization behavior caused by local adiabatic effects”. This process effectively transitions into a thermal deformation process. Under thermal activation, the microstructure undergoes rapid dynamic recovery and recrystallization. This phenomenon occurs during CR deformation, and its recrystallization mechanism is consistent with dynamic recrystallization during hot deformation. Additionally, Σ3 {120} type non-coherent twins were formed in the recrystallized grains. Understanding the evolution of the microstructure and mechanical behavior during the plastic deformation of electrolytic nickel is crucial for controlling and optimizing short-process rolling and even for improving the performance of nickel strips.

## Figures and Tables

**Table 1:** Main Chemical Components of Electrolytic Nickel (wt.%) [27].

Co	C	Si	P	S	Fe	Cu	Al	Mg	Ni
0.0241	0.0120	0.0298	<0.0003	<0.0003	0.0082	0.0033	<0.0001	<0.0001	Bal.

## Data Availability

The original contributions presented in this study are included in the article. Further inquiries can be directed to the corresponding authors.
